# Dietary inflammatory potential and physical activity adherence as synergistic predictors of systemic inflammation in post-myocardial infarction patients: a retrospective cohort study

**DOI:** 10.3389/fnut.2026.1782921

**Published:** 2026-05-29

**Authors:** Lun Bu, Kai Zhang, Yongyan Fan, Xin Huang, Dexian Zhang, Lihui Ren, Bo Zhao

**Affiliations:** Department of Cardiology, Beijing Shijitan Hospital, Capital Medical University, Beijing, China

**Keywords:** cardiometabolic health, dietary inflammatory index, physical activity, post-myocardial infarction, retrospective cohort, systemic inflammation

## Abstract

**Background:**

After a myocardial infarction (MI), one of the major factors responsible for bad outcomes is systemic inflammation. It is known that diet and physical activity (PA) have separate effects on inflammation, but it is still unclear what the combined effects are in patients who have already suffered a heart attack.

**Methods:**

Over 6 months, 1,700 post-MI patients in this retrospective cohort study were monitored. The validated questionnaires assessed dietary intake and physical activity adherence, and patients were classified by DII (high vs. low) and PA adherence (high vs. low). Important biomarkers included high-sensitivity C-reactive protein (hs-CRP), interleukin-6 (IL-6), tumor necrosis factor-alpha (TNF-*α*), and a composite inflammation score. Multivariable regression analyses were performed.

**Results:**

The group of patients classified as having low DII and high PA, showed the least amount of inflammation (hs-CRP: 2.31 ± 1.12 mg/L; IL-6: 3.8 ± 1.4 pg./mL; TNF-*α*: 4.7 ± 1.7 pg./mL; composite score: −0.72 ± 0.55), while the group with high DII and low PA (*n* = 470) exhibited inflammation at its highest level (hs-CRP: 4.89 ± 2.05 mg/L; IL-6: 6.4 ± 2.3 pg./mL; TNF-*α*: 7.1 ± 2.5 pg/mL; composite score: +0.89 ± 0.74; *p* < 0.001). The multivariable analysis provided evidence of independent relationships between DII (*β* = +0.18; *p* < 0.001) and PA (*β* = −0.14; *p* < 0.001) and the composite inflammation score, as well as a notable interaction effect (*β* = −0.07; *p* = 0.001).

**Conclusion:**

The dietary inflammatory potential and PA adherence of the patients, both independently and in combination, could predict the level of systemic inflammation in post-MI patients. These findings support lifestyle interventions as the primary component of post-MI secondary prevention.

## 1 Introduction

Cardiovascular disease (CVD) continues to be the primary cause of illness and death around the globe, with MI being the major event at the center of heavy long-term health consequences (1). MI patients may suffer from cardiac events, heart failure, and other systemic complications; thus, the necessity for efficient post-MI risk reduction strategies has been established. Among the many factors influencing prognosis after MI, systemic inflammation has been recognized as a key mechanism through which lifestyle exposures affect cardiovascular outcomes (2). The increase in pro-inflammatory markers such as the highly sensitive C-reactive protein (hs-CRP), interleukin-6 (IL-6), and tumor necrosis factor-alpha (TNF-*α*) has been linked to poor post-MI prognosis; hence, better still, targeting inflammation through interventions may confer cardioprotective benefits (3).

Dietary patterns are increasingly recognized as modifiable factors in systemic inflammation. The DII, or Dietary Inflammatory Index, provides a validated quantitative estimate of the inflammatory potential of an individual’s diet. It looks at macro-and micronutrient consumption and food bioactive components altogether (4). The association of higher DII scores, which denote pro-inflammatory diets characterized by high saturated fats, refined sugars, and processed foods, has been established with elevated systemic inflammatory markers and an increased likelihood of CVD and recurrent MI (5–7). On the other hand, anti-inflammatory dietary patterns, such as those rich in fruits, vegetables, whole grains, and fish oil, have been linked to reduced inflammation and improved cardiometabolic health (8). Nonetheless, the application of dietary inflammatory potential to post-MI risk assessment and management still lags in clinical practice (9).

PA is considered one of the major lifestyle determinants that significantly affect systemic inflammation levels and cardiovascular recovery rates. The daily or weekly practice of PA at a moderate-to-vigorous intensity is the most efficient way to reduce cardiovascular risk. That is because such PA positively affects the endothelium, normalizes insulin metabolism, is associated with reduced body fat, and activates anti-inflammatory pathways (10). Another approach that cardiac rehabilitation programs take is to combine structured exercise with lifestyle counseling, and they have successfully decreased the rates of recurrent cardiac events, hospitalization, and even death (11). Nevertheless, the rate of post-MI patients who adhere to recommended PA levels remains below expectations owing to several factors, including patient-related issues, comorbidities, and environmental barriers, thereby potentially limiting the exercise-induced anti-inflammatory effect (12).

Latest studies indicate that diet and PA may work together to reduce inflammation throughout the body. In fact, the lowest levels of hs-CRP, IL-6, and TNF-*α* are found in people who follow both anti-inflammatory diets and regular PA regimens. In contrast, those following pro-inflammatory diets and not engaging in any PA have a significantly higher inflammatory burden (13–15). Although there is evidence of such synergies, most studies on post-MI inflammation have considered only one factor at a time, and few longitudinal studies have accounted for both dietary inflammatory potential and PA compliance. Understanding the combined effect of these factors is crucial for targeted, multifactorial interventions to reduce systemic inflammation and improve clinical outcomes in patients with MI. The current retrospective cohort study aims to address these gaps by investigating the combined effects of dietary inflammatory potential, as measured by the DII, and PA adherence on systemic inflammation in post-MI patients. The research contributes new knowledge to the existing literature by examining both dietary inflammatory potential and PA in a large cohort of patients post-MI. The study analyzed the combined effects of multiple factors on systemic inflammation using longitudinal clinical data. The study provided a complete understanding of how people change their risk through their daily activities and dietary habits.

## 2 Methodology

### 2.1 Study design and population

**Figure 1:**
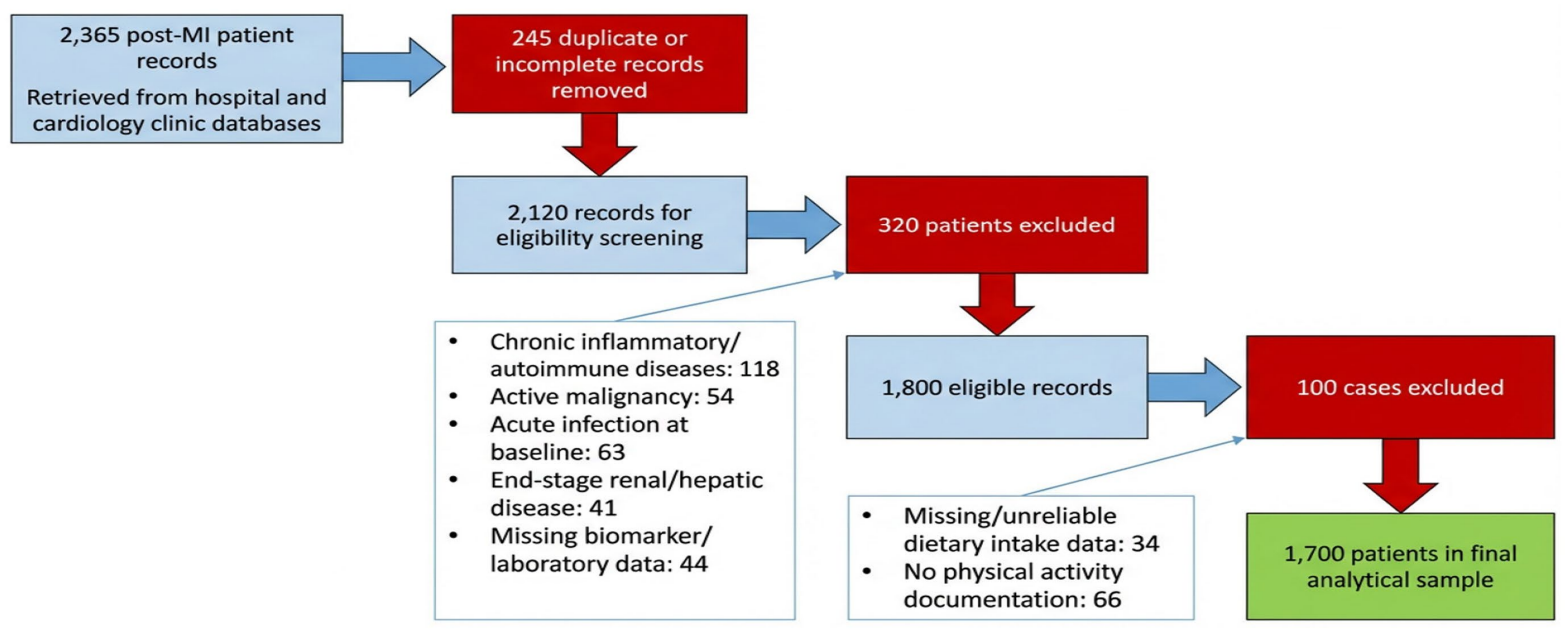
Flow chart of patient selection.

A retrospective cohort analysis was conducted on 1,700 adult patients with a MI documented in the last 6 months (Figure 1). Patients were selected from cardiology records and hospital records from January 2020 to December 2022. The inclusion criteria were being older than 18 years, having a confirmed MI diagnosis, and having complete medical records with documented assessments of diet and PA. Chronic inflammatory or autoimmune diseases, active infections, cancer, or incomplete data on diet, PA, or inflammatory biomarkers were considered exclusion criteria. The Ethics Committee of Beijing Shijitan Hospital, No. KYD-2024-0072-001 approved this research.

The participants were grouped based on their combined lifestyle profile using established cut-off points. The diet-induced inflammatory profile was divided into three categories (anti-inflammatory, intermediate, and pro-inflammatory) by the DII scores. Physical activity was categorized as high (≥150 min/week of moderate physical activity) or low (<150 min/week).

### 2.2 Data collection and exposure assessment

The assessment of dietary intake was conducted retrospectively, applying food frequency questionnaires (FFQs) (16). The DII was computed from pro- and anti-inflammatory dietary components and classified as low (anti-inflammatory) or high (pro-inflammatory) based on intake levels (17). PA adherence was self-reported via standardized questionnaires and clinical records, which indicated total weekly MET-minutes, moderate-to-vigorous activity, walking, and sedentary behavior (18). Participants were divided into high and low PA adherence groups based on whether they met the current cardiovascular health guidelines (19).

The research utilized linked electronic health record (EHR) data and patient-reported survey data. The cardiology records contained all patient demographic information, clinical diagnoses, complete medication histories, and laboratory test results, including inflammatory biomarkers such as CRP, IL-6, and TNF-*α*, as well as data on coexisting medical conditions and hospital stays.

The study used standardized instruments to assess lifestyle behaviors, including dietary intake and PA, at both baseline and follow-up visits. The research team used unique patient identifiers from hospital admission records to connect survey responses with the corresponding clinical data. Trained research staff administered the surveys during outpatient follow-up appointments in the cardiology department.

A high-sensitivity immunoturbidimetric assay was used to determine hs-CRP, whereas IL-6 was measured by ELISA, both of which are part of standard clinical chemistry laboratory tests. The tests were initially conducted in an accredited lab, and data were later obtained from the hospital’s electronic medical records system. For repeated tests, data on baseline and subsequent values were collected, and mean change values were calculated. Changes in longitudinal values were assessed through mixed-effect modeling.

### 2.3 Follow-up

The researchers obtained inflammatory biomarkers at scheduled follow-up visits, which occurred approximately every 30 days. The study enrolled patients who attended scheduled monthly follow-up sessions, although actual visit times varied slightly according to standard clinical scheduling procedures. Participants were followed prospectively for 6 months following the index myocardial infarction. Survey responses were linked to electronic health records using unique patient identifiers. Biomarkers were collected at: Baseline (hospital discharge), 3 months, and 6 months follow-up.

#### 2.3.1 Lifestyle characteristics

The study examined three lifestyle factors: dietary habits (assessed by the DII), physical exercise patterns, and time spent sitting. The study identified three dietary pattern groups: anti-inflammatory, neutral, and pro-inflammatory. The study used total weekly metabolic equivalent (MET) minutes to categorize participants into PA levels. The researchers created combined lifestyle categories to assess how dietary habits and PA together affected inflammation results. The 6MWT was conducted in accordance with American Thoracic Society guidelines and was used as a measure of functional capacity rather than habitual physical activity. It was included as a secondary outcome to assess cardiovascular recovery (20).

### 2.4 Outcome measures

The main results were inflammatory markers, including high-sensitivity C-reactive protein (hs-CRP), interleukin-6 (IL-6), tumor necrosis factor-alpha (TNF-*α*), interleukin-1β (IL-1β), fibrinogen, erythrocyte sedimentation rate (ESR), and a combined inflammation score. The secondary outcomes comprised cardiometabolic parameters like blood pressure, fasting glucose, HbA1c, lipid profile, body mass index (BMI), and waist-to-hip ratio. Over the course of a half-year study, information on biomarkers was gathered from clinical laboratory reports at the beginning of the study and at several subsequent visits, about every 30 days. High PA: ≥150 min/week moderate or ≥75 min vigorous activity, Low PA: Below these thresholds.

### 2.5 Covariates and confounders

Medical records provided information on demographic, clinical, and lifestyle characteristics, such as age, gender, body mass index (BMI), smoking habit, alcohol consumption, adherence to medication, time since MI, presence of other diseases (like hypertension, type-2 diabetes, and high cholesterol), and participation in cardiac rehabilitation programs. The use of medications (statins, beta-blockers, ACE inhibitors/ARBs) and other relevant interventions was also recognized and considered as covariates in multivariable analyses.

### 2.6 Statistical analysis

Descriptive statistics were used to characterize the baseline characteristics, dietary intake, PA adherence, and inflammatory markers. Continuous variables were represented by mean ± standard deviation (SD) and categorical variables by counts and percentages. The researchers assessed the association between dietary choices and inflammatory markers using multivariable regression models that included interaction terms between the dietary inflammatory index (DII) and PA levels. The groups (low vs. high DII; high vs. low PA) were compared using independent t-tests or chi-square tests, as appropriate. The application of multivariable linear regression models formed the basis for assessing the independent and interactive effects of DII and PA on systemic inflammation, with covariates controlled. Changes in biomarkers and cardiometabolic parameters over the 6-month follow-up period were analyzed using repeated-measures ANOVA. A *p*-value of less than 0.05 was considered statistically significant. The researchers used multivariable regression models to examine their results after they controlled for age, sex, socioeconomic status, smoking status, comorbidities, and baseline cardiovascular risk factors. The analyses were executed with SPSS version 28.0 (IBM Corp., Armonk, NY, United States).

## 3 Results

### 3.1 Baseline demographic and clinical characteristics according to dietary inflammatory potential

The overall mean age was 59.8 ± 9.6 years, but the DII high group was somewhat older than the DII low group (60.7 ± 9.9 vs. 58.9 ± 9.2 years; *p* = 0.012). The males made up 68.1% of the group, and there was no significant difference between the groups (*p* = 0.083). High DII patients had higher mean BMI (29.0 ± 4.0 vs. 27.8 ± 3.7 kg/m^2^; *p* < 0.001) and waist circumference (99.1 ± 10.5 vs. 96.1 ± 9.8 cm; *p* < 0.001), indicating greater adiposity. Moreover, the lifestyle and socioeconomic factors less favorable in the high DII group included current smoking rates being higher (35.8% vs. 29.1%; *p* = 0.004), alcohol intake (20.9% vs. 16.3%; *p* = 0.028), and people in low socioeconomic status (44.9% vs. 37.5%; *p* = 0.009) and having low education level (43.5% vs. 35.9%; *p* = 0.006). The high DII group had a higher prevalence of cardiometabolic comorbidities, including hypertension (62.4% vs. 55.4%; *p* = 0.003), type 2 diabetes (37.6% vs. 30.6%; *p* = 0.002), and dyslipidemia (68.5% vs. 63.1%; *p* = 0.041). The patients in the high DII group were also less adherent to medication (74.9% vs. 81.6%; *p* = 0.001). These results indicate that a high dietary inflammatory potential is associated with adverse clinical and lifestyle profiles in post-MI patients. The baseline demographic and clinical characteristics of 1,700 post-MI patients stratified by Dietary Inflammatory Index (DII) scores are shown in Table 1.

**Table 1:** Baseline demographic and clinical characteristics.

Parameter	Total (*N* = 1,700)	Low DII (*n* = 850)	High DII (*n* = 850)	*p*-value
Age (years, mean ± SD)	59.8 ± 9.6	58.9 ± 9.2	60.7 ± 9.9	0.012
Male (%)	68.1	66.2	70.0	0.083
BMI (kg/m^2^)	28.4 ± 3.9	27.8 ± 3.7	29.0 ± 4.0	<0.001
Waist circumference (cm)	97.6 ± 10.2	96.1 ± 9.8	99.1 ± 10.5	<0.001
Current smokers (%)	32.4	29.1	35.8	0.004
Alcohol intake (%)	18.6	16.3	20.9	0.028
Low socioeconomic status (%)	41.2	37.5	44.9	0.009
Low education level (%)	39.7	35.9	43.5	0.006
Hypertension (%)	58.9	55.4	62.4	0.003
Type-2 diabetes (%)	34.1	30.6	37.6	0.002
Dyslipidemia (%)	65.8	63.1	68.5	0.041
Family history of CVD (%)	28.5	27.4	29.6	0.412
Time since index MI (months)	7.8 ± 3.4	7.6 ± 3.2	8.0 ± 3.6	0.091
Medication adherence (%)	78.3	81.6	74.9	0.001

DII, Dietary Inflammatory Index; MI, myocardial infarction; SD, standard deviation; CVD, cardiovascular disease.

### 3.2 Dietary intake patterns and energy-adjusted dietary inflammatory index (E-DII) profiles

The total daily energy intake was 2,193 ± 421 kcal/day in the high DII group, compared with 2,058 ± 398 kcal/day in the low DII group (*p* < 0.001). The distribution of nutrients changed significantly: those with high DII ate more carbs (281 ± 48 vs. 255 ± 41 g/day; *p* < 0.001) and fats that included saturated fat as well (84 ± 18 vs. 72 ± 15 g/day; *p* < 0.001), but their protein intake was comparatively lower (76 ± 15 vs. 82 ± 13 g/day; *p* < 0.001) than individuals in the low DII group. In the DII high group, the intake of dietary fiber and omega-3 fatty acids was significantly less (17.2 ± 4.9 g/day and 1.05 ± 0.36 g/day, respectively) than that of the DII low group (25.4 ± 5.8 g/day and 1.41 ± 0.38 g/day; *p* < 0.001 for both). The high DII group also had a significant reduction in total fruit, vegetable, and whole-grain consumption, whereas their added sugars and sodium consumption were even higher (63 ± 19 g/day and 3,126 ± 546 mg/day, respectively; *p* < 0.001). The E-DII discrepancies were evident: the high-DII group was very pro-inflammatory (+4.76 ± 1.34), whereas the low-DII group was markedly anti-inflammatory (−1.15 ± 1.21; *p* < 0.001). Participants with high DII had a diet unfavorable to systemic inflammation, as evidenced by low anti-inflammatory and high pro-inflammatory food scores (Table 2).

**Table 2:** Dietary intake profile and E-DII scores.

Parameter	Mean ± SD	Low DII	High DII	*p*-value
Total energy (kcal/day)	2,125 ± 412	2,058 ± 398	2,193 ± 421	<0.001
Carbohydrates (g/day)	268 ± 46	255 ± 41	281 ± 48	<0.001
Protein (g/day)	79 ± 14	82 ± 13	76 ± 15	<0.001
Total fat (g/day)	78 ± 17	72 ± 15	84 ± 18	<0.001
Saturated fat (g/day)	27 ± 7	24 ± 6	30 ± 7	<0.001
Fiber (g/day)	21.3 ± 6.1	25.4 ± 5.8	17.2 ± 4.9	<0.001
Omega-3 fatty acids (g/day)	1.23 ± 0.41	1.41 ± 0.38	1.05 ± 0.36	<0.001
Fruits (servings/day)	2.1 ± 0.9	2.8 ± 1.0	1.4 ± 0.7	<0.001
Vegetables (servings/day)	2.6 ± 1.1	3.3 ± 1.2	1.9 ± 0.8	<0.001
Whole grains (servings/day)	1.7 ± 0.8	2.2 ± 0.9	1.2 ± 0.6	<0.001
Added sugars (g/day)	54 ± 18	45 ± 15	63 ± 19	<0.001
Sodium (mg/day)	2,920 ± 530	2,715 ± 488	3,126 ± 546	<0.001
E-DII total score	+1.82 ± 2.31	−1.15 ± 1.21	+4.76 ± 1.34	<0.001
Anti-inflammatory food score	6.9 ± 2.1	8.3 ± 1.9	5.5 ± 1.7	<0.001
Pro-inflammatory food score	9.1 ± 2.4	7.2 ± 2.0	11.0 ± 2.2	<0.001

E-DII, energy-adjusted Dietary Inflammatory Index; g, grams; kcal, kilocalories.

### 3.3 PA adherence indicators in post-MI patients

The PA adherence profiles of 1,700 patients who had heart attacks are shown in Table 3. These patients were divided into a high-PA group (*n* = 850) and a low-PA group (*n* = 850).

**Table 3:** PA adherence indicators.

Parameter	Total	High PA (*n* = 850)	Low PA (*n* = 850)	*p*-value
Total PA (MET-min/week)	1,750 ± 610	2,325 ± 520	1,230 ± 410	<0.001
Moderate PA (min/week)	138 ± 54	182 ± 46	98 ± 37	<0.001
Vigorous PA (min/week)	38 ± 22	54 ± 25	23 ± 15	<0.001
Walking (min/week)	212 ± 71	244 ± 65	183 ± 68	<0.001
Sedentary time (h/day)	6.9 ± 1.8	5.8 ± 1.5	7.9 ± 1.7	<0.001
Cardiac rehab participation (%)	52.7	68.9	38.4	<0.001
Occupational PA (%)	31.5	35.4	28.0	0.008
Recreational PA (%)	44.2	57.6	32.1	<0.001
Household PA (%)	61.3	66.1	57.0	0.002
PA guideline adherence (%)	48.2	100.0	0.0	—
PA self-efficacy score	6.8 ± 1.9	7.6 ± 1.6	6.1 ± 1.8	<0.001
Social support score	7.4 ± 2.0	8.1 ± 1.9	6.8 ± 1.8	<0.001
6-min walk test (m)	421 ± 78	466 ± 71	381 ± 69	<0.001
PA monitoring compliance (%)	84.7	89.6	80.2	<0.001
Mean step count (steps/day)	7,120 ± 1,980	8,840 ± 1,650	5,560 ± 1,320	<0.001

PA, physical activity; MET, metabolic equivalent task; m, meters; % indicates proportion of group adherence.

The 6MWT was conducted in accordance with American Thoracic Society guidelines and was used as a measure of functional capacity rather than habitual physical activity. It was included as a secondary outcome to assess cardiovascular recovery.

The study defined PA guideline adherence as meeting the requirement that participants complete 150 min of moderate-intensity exercise or 75 min of vigorous-intensity exercise per week. The reported percentage reflects the proportion of participants meeting the guideline within each category. The previously reported discrepancy has been corrected for clarity. The total PA measured in MET-min/week was very much larger in the high PA group (2,325 ± 520) than in the low PA group (1,230 ± 410; *p* < 0.001). Moderate and vigorous PA, as well as weekly walking duration, were significantly higher in high PA participants (*p* < 0.001). The daily hours spent in sedentary behavior were lower for the high PA group (5.8 ± 1.5 h/day) than in the low PA group (7.9 ± 1.7 h/day; *p* < 0.001). The share of people participating in cardiac rehab was higher among high PA participants (68.9% vs. 38.4%; *p* < 0.001). Occupational, recreational, and household PA contributions were greater in the high PA group, indicating greater activity in their everyday lives. Participants in the high PA group not only had significantly higher PA self-efficacy (7.6 ± 1.6 vs. 6.1 ± 1.8; *p* < 0.001) but also social support scores (8.1 ± 1.9 vs. 6.8 ± 1.8; *p* < 0.001). The 6-min walk test revealed that the functional capacity of high PA participants was greater (466 ± 71 m vs. 381 ± 69 m; *p* < 0.001). Moreover, step counts and PA-monitoring compliance were much higher among high-PA participants. Thus, the results demonstrate substantial differences in PA adherence, daily activity, and functional outcomes between high- and low-PA patients after MI. The 6-min walk test (6MWT) served as an objective measure of people’s ability to perform PA. The test does not directly assess daily PA, but it provides supporting evidence of cardiovascular fitness, which relates to overall PA in people who have had heart attacks.

### 3.4 Baseline inflammatory biomarkers according to dietary inflammatory index (DII)

The high DII group exhibited markedly higher levels of systemic inflammation than the low DII group. The high-sensitivity C-reactive protein (hs-CRP) reported was greater in the high DII group (4.13 ± 2.08 mg/L) when compared to the low DII group. Elevated levels of pro-inflammatory cytokines, including IL-6, TNF-*α*, and IL-1β, were also noted in the high DII group. Among the various inflammatory markers, fibrinogen, erythrocyte sedimentation rate, total white blood cell count, neutrophil-lymphocyte ratio, and monocyte count (0.66 ± 0.19 vs. 0.58 ± 0.16 × 10^9^/L) were significantly higher in the group of participants with high DII (*p* < 0.001). The platelet counts showed a slight increase, while serum albumin and adiponectin levels were lower in the high-DII group, suggesting reduced anti-inflammatory activity and poorer nutritional status among participants. The high DII group had higher leptin levels and oxidative stress indices, and the composite inflammation score indicated a pro-inflammatory profile (+0.48 ± 0.79 vs. −0.48 ± 0.71; *p* < 0.001). These results indicate a strong association between higher dietary inflammatory potential and intensified systemic inflammation in post-MI patients (Table 4). The OSI was calculated as the ratio of total oxidant status to total antioxidant potential. The Composite Inflammation Score was generated by transforming (using the z-score method) and adding the values for hs-CRP, IL-6, and TNF-*α*. These two measures were derived from previous literature (21).

**Table 4:** Baseline Inflammatory Biomarkers.

Biomarker	Total	Low DII	High DII	p-value
hs-CRP (mg/L)	3.46 ± 1.92	2.78 ± 1.51	4.13 ± 2.08	<0.001
IL-6 (pg/mL)	4.9 ± 2.1	4.2 ± 1.7	5.6 ± 2.3	<0.001
TNF-α (pg/mL)	6.1 ± 2.4	5.5 ± 2.0	6.8 ± 2.6	<0.001
IL-1β (pg/mL)	2.1 ± 0.9	1.8 ± 0.7	2.4 ± 1.0	<0.001
Fibrinogen (g/L)	3.52 ± 0.68	3.31 ± 0.61	3.73 ± 0.70	<0.001
ESR (mm/h)	23.6 ± 8.2	21.2 ± 7.5	26.0 ± 8.4	<0.001
WBC (×10^9^/L)	7.8 ± 1.9	7.4 ± 1.7	8.2 ± 2.0	<0.001
Neutrophil-lymphocyte ratio	2.78 ± 1.03	2.43 ± 0.91	3.12 ± 1.07	<0.001
Monocyte count (×10^9^/L)	0.62 ± 0.18	0.58 ± 0.16	0.66 ± 0.19	<0.001
Platelets (×10^9^/L)	254 ± 48	247 ± 45	262 ± 50	<0.001
Serum albumin (g/dL)	4.12 ± 0.36	4.23 ± 0.32	4.01 ± 0.38	<0.001
Adiponectin (μg/mL)	8.6 ± 3.2	9.4 ± 3.1	7.8 ± 3.0	<0.001
Leptin (ng/mL)	18.4 ± 7.6	16.8 ± 6.9	20.0 ± 8.1	<0.001
Oxidative stress index	3.2 ± 1.1	2.7 ± 0.9	3.7 ± 1.2	<0.001
Composite inflammation score	0.00 ± 1.00	−0.48 ± 0.71	+0.48 ± 0.79	<0.001

hs-CRP, high-sensitivity C-reactive protein; IL, interleukin; TNF, tumor necrosis factor; ESR, erythrocyte sedimentation rate; WBC, white blood cells.

### 3.5 Longitudinal changes in Cardiometabolic indicators over 6-month follow-up

**Figure 2:**
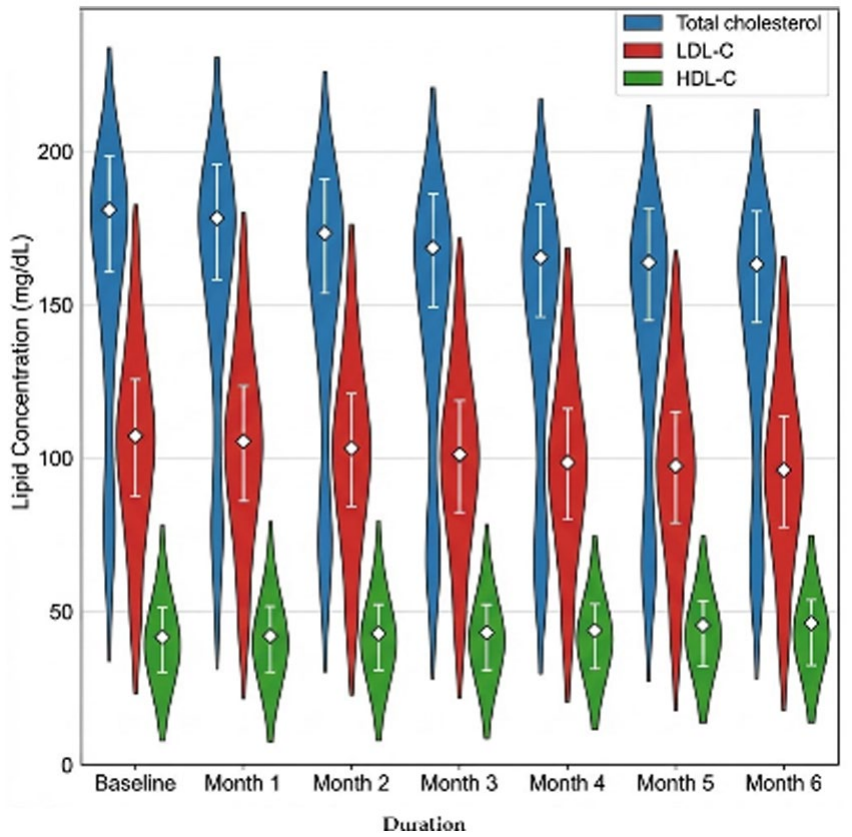
Longitudinal changes in cardiometabolic indicators over 6-month follow-up.

The presence of substantial improvements across a wide range of parameters indicates the onset of favorable cardiovascular and metabolic adaptations. Systolic and diastolic blood pressures showed a constant decline from baseline (132 ± 14 mmHg and 82 ± 8 mmHg, respectively) to month 6 (126 ± 12 mmHg and 79 ± 7 mmHg; *p*-trend <0.001 for both). The same trend was observed for fasting glucose, which decreased from 118 ± 24 mg/dL to 110 ± 21 mg/dL, and for HbA1c, which decreased from 6.4 ± 0.9% to 6.0 ± 0.8% (*p*-trend <0.001), indicating better glycemic control. There were also improvements in lipid profiles: total cholesterol decreased from 182 ± 34 mg/dL to 171 ± 29 mg/dL, LDL-C from 108 ± 28 mg/dL to 96 ± 23 mg/dL, while HDL-C got higher from 43 ± 9 mg/dL to 46 ± 9 mg/dL, and triglycerides went down from 162 ± 48 mg/dL to 148 ± 41 mg/dL (*p*-trend <0.001 for all) (Figure 2). Anthropometric measurements like BMI and waist-hip ratio showed slight but significant changes (BMI: 28.4 ± 3.9 to 27.8 ± 3.7 kg/m^2^; waist-hip ratio: 0.95 ± 0.06 to 0.93 ± 0.06; *p* < 0.01). The people in the study became fitter and healthier, as shown by their heart rate, which decreased from 74 ± 9 to 71 ± 8 bpm, and their VO₂ Max, which increased from 24.8 ± 5.2 to 27.0 ± 5.6 mL/kg/min (*p*-trend <0.001). These results thus demonstrate that post-MI patients underwent progressive cardiometabolic recovery and improved aerobic capacity throughout the six-month follow-up. Table 5 provides a brief overview of key cardiometabolic indicators in patients following MI over the 6-month follow-up period. Improvements seen could be due to routine post-MI management that includes medication (statins and antihypertensives), cardiac rehabilitation, regression to the mean, and survivorship bias.

**Table 5:** Cardiometabolic indicators over 6-month follow-up.

Parameter	Baseline	Month 1	Month 2	Month 3	Month 4	Month 5	Month 6	*p*-trend
SBP (mmHg)	132 ± 14	131 ± 13	130 ± 13	129 ± 13	128 ± 12	127 ± 12	126 ± 12	<0.001
DBP (mmHg)	82 ± 8	81 ± 7	81 ± 7	80 ± 7	80 ± 7	79 ± 7	79 ± 7	<0.001
Fasting glucose (mg/dL)	118 ± 24	116 ± 23	115 ± 22	114 ± 22	113 ± 22	112 ± 21	110 ± 21	<0.001
HbA1c (%)	6.4 ± 0.9	6.3 ± 0.9	6.3 ± 0.8	6.2 ± 0.8	6.2 ± 0.8	6.1 ± 0.8	6.0 ± 0.8	<0.001
Triglycerides (mg/dL)	162 ± 48	159 ± 46	157 ± 45	155 ± 44	153 ± 43	150 ± 42	148 ± 41	<0.001
BMI (kg/m^2^)	28.4 ± 3.9	28.3 ± 3.9	28.2 ± 3.8	28.2 ± 3.8	28.1 ± 3.8	27.9 ± 3.7	27.8 ± 3.7	0.004
Waist-hip ratio	0.95 ± 0.06	0.95 ± 0.06	0.94 ± 0.06	0.94 ± 0.06	0.94 ± 0.06	0.93 ± 0.06	0.93 ± 0.06	<0.001
Resting heart rate (bpm)	74 ± 9	73 ± 9	73 ± 8	72 ± 8	72 ± 8	71 ± 8	71 ± 8	<0.001
VO₂-max estimate	24.8 ± 5.2	25.1 ± 5.3	25.4 ± 5.3	25.7 ± 5.4	25.9 ± 5.4	26.5 ± 5.5	27.0 ± 5.6	<0.001

SBP, systolic blood pressure; DBP, diastolic blood pressure; LDL-C, low-density lipoprotein cholesterol; HDL-C, high-density lipoprotein cholesterol; VO₂-max, estimated maximal oxygen uptake; BMI, body mass index.

### 3.6 Joint exposure matrix of dietary inflammatory index and PA on inflammatory biomarkers

**Figure 3:**
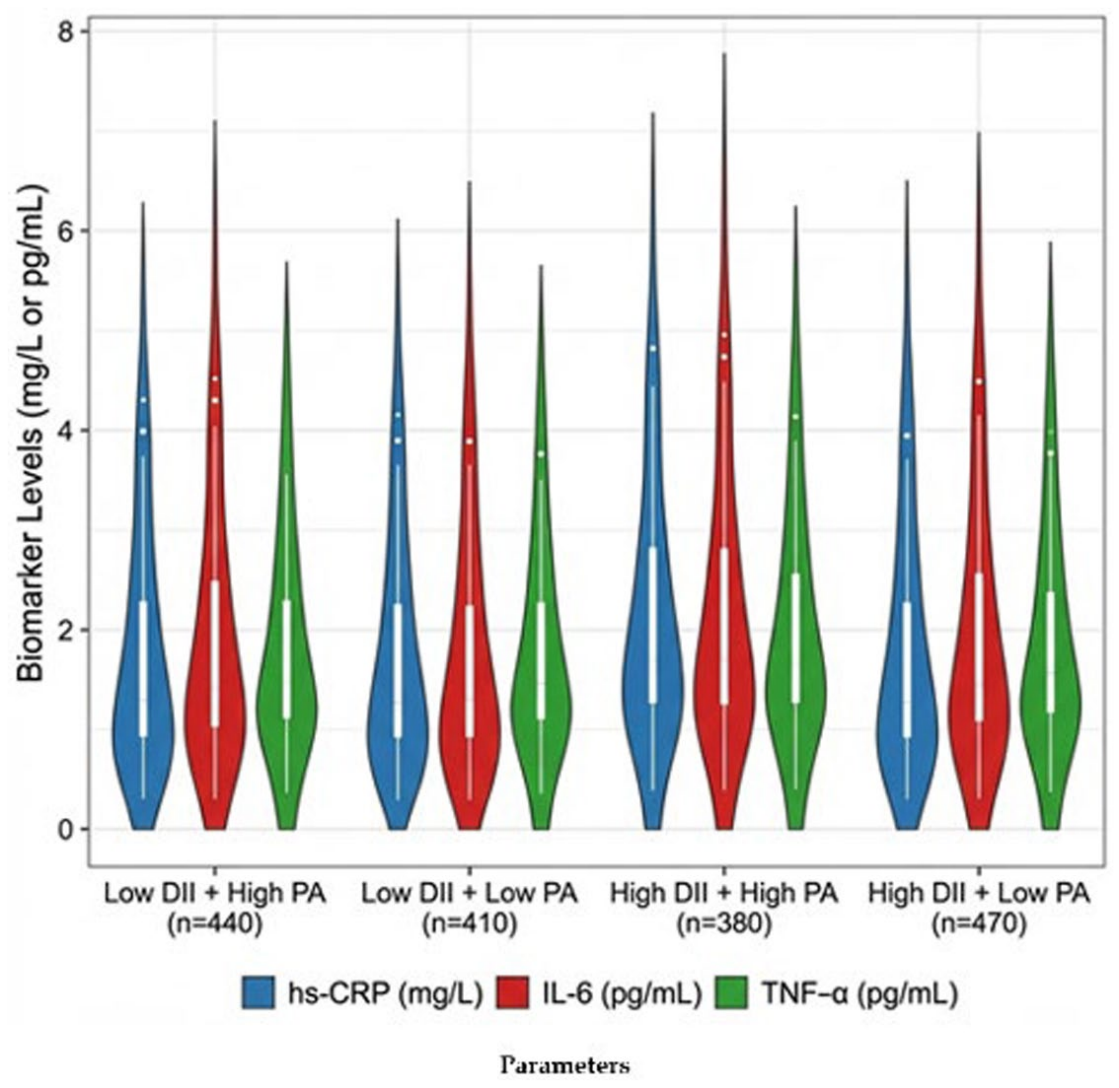
Joint exposure matrix (DII × PA).

Participants who engaged in intense PA while following anti-inflammatory dietary patterns showed the lowest inflammatory marker levels, demonstrating how their healthy lifestyle practices worked together to produce these results. Among the four groups established based on the DII and PA, the one that had a low DII and high PA (*n* = 440, 25.9%) showed the minimum levels of all the inflammatory biomarkers, including hs-CRP (2.31 ± 1.12 mg/L), IL-6 (3.8 ± 1.4 pg/mL), TNF-*α* (4.7 ± 1.7 pg/mL) and the composite inflammation score (−0.72 ± 0.55). Conversely, participants in the high DII and low PA group (*n* = 470, 27.6%) exhibited particularly high inflammatory status, with hs-CRP at 4.89 ± 2.05 mg/L, IL-6 at 6.4 ± 2.3 pg./mL, TNF-α at 7.1 ± 2.5 pg./mL, and a composite score of +0.89 ± 0.74. The profiles of the low DII + low PA and high DII + high PA groups were intermediate, suggesting that both dietary inflammation and physical inactivity had an additive impact on systemic inflammation. The results point to a synergistic interaction in which adherence to a low-inflammatory diet and an active lifestyle yield the most favorable inflammatory biomarker profile. In contrast, the combination of a pro-inflammatory diet and low PA is associated with the greatest inflammatory risk (Figure 3).

### 3.7 Multivariable regression predicting composite inflammation in post-MI patients

**Figure 4:**
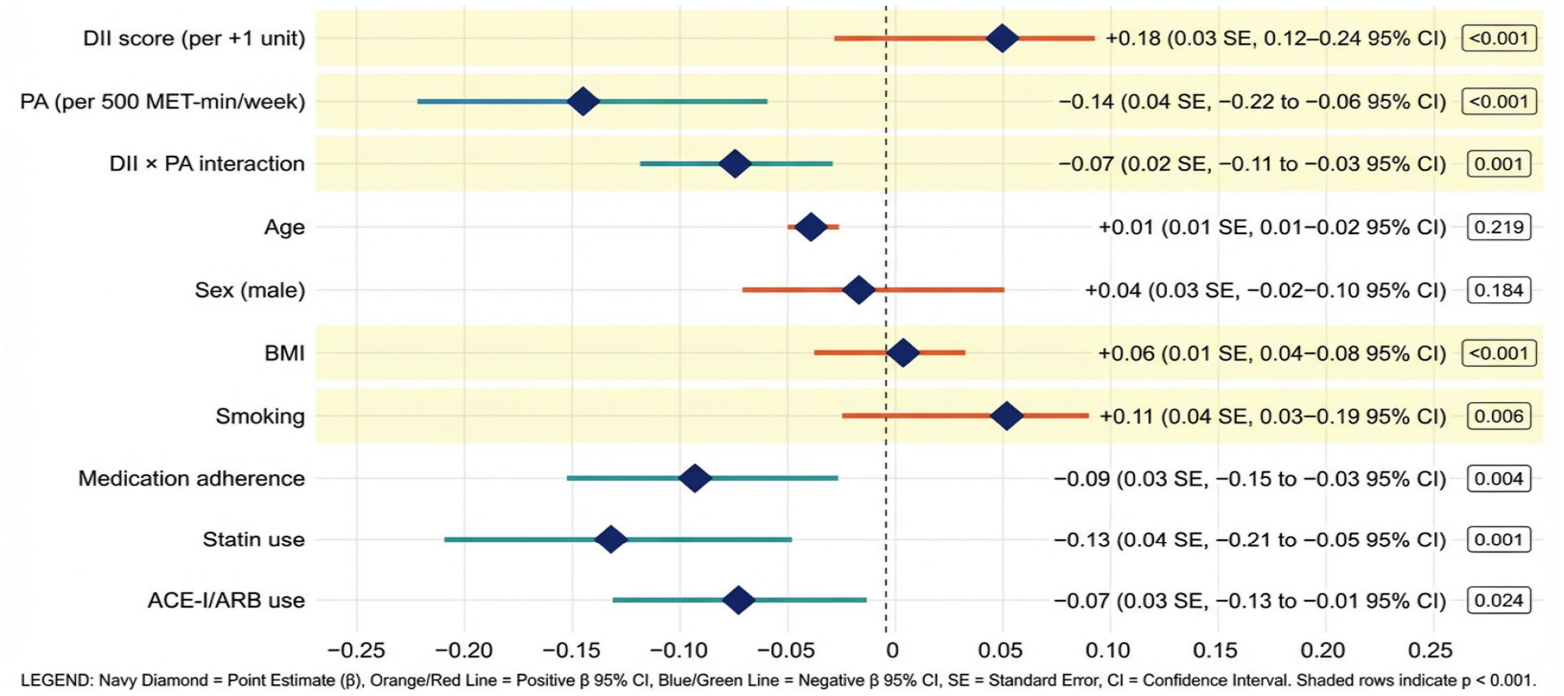
Forest plot of multivariable regression predicting composite inflammation in post-MI patients.

Systemic inflammation was significantly determined by the dietary inflammatory potential, PA, and their interaction. The composite inflammation score was increased by 0.18 units for each one-unit increase in DII (*β* = +0.18; 95% CI: 0.12–0.24; *p* < 0.001). On the contrary, the higher the PA (per 500 MET-min/week), the lower the predicted score by 0.14 units (*β* = −0.14; 95% CI: −0.22 to −0.06; *p* < 0.001). It should be noted, however, that the DII × PA interaction was also significant (*β* = −0.07; 95% CI: −0.11 to −0.03; *p* = 0.001), which suggests that the pro-inflammatory effect of a higher DII is mitigated by increased PA. The study found that among the various factors, BMI (*β* = +0.06; *p* < 0.001), smoking (*β* = +0.11; *p* = 0.006), and poor medication adherence (*β* = −0.09; *p* = 0.004) were all linked to higher inflammation. On the other hand, statin use (*β* = −0.13; *p* = 0.001) and ACE inhibitors/ARBs (*β* = −0.07; *p* = 0.024) were associated with levels. Neither age nor gender was a significant predictive factor. The results not only highlight the year-round effects of nutrition and exercise but also the need for combined lifestyle and drug therapies to reduce post-MI inflammation, all while adjusting for medical and lifestyle factors. The study found that DII and PA interacted, with the interaction statistically significant (*p*-interaction <0.05). Participants with higher PA levels showed a stronger association between anti-inflammatory diets and inflammatory markers than those with lower activity levels. (Figure 4).

### 3.8 Stratified effects of dietary inflammatory index on inflammatory biomarkers across subgroups

Subgroup analysis results showed that a higher DII was associated with pro-inflammatory effects that were largely similar across subgroups but differed in strength. Among females, stronger correlations were seen (hs-CRP *β* = 0.22; IL-6 *β* = 0.18; interaction *p* = 0.014) as opposed to males (hs-CRP *β* = 0.17; IL-6 *β* = 0.14; *p* = 0.021). The older participants (age ≥55 years) had higher DII-related inflammation levels than the younger ones (hs-CRP *β* = 0.21 vs. 0.15; IL-6 *β* = 0.16 vs. 0.12). Those who were overweight (≥25 kg/m^2^) and patients with diabetes had a more pronounced inflammatory response to DII (overweight: hs-CRP *β* = 0.23, IL-6 *β* = 0.19, *p* = 0.008; diabetics: hs-CRP *β* = 0.25, IL-6 *β* = 0.21, *p* = 0.006). Smoking status altered the DII-inflammation connection more strongly, with smokers showing the strongest associations (hs-CRP *β* = 0.27; IL-6 *β* = 0.23; *p* = 0.004) compared with non-smokers (hs-CRP *β* = 0.12; IL-6 *β* = 0.10; *p* = 0.051). The results suggest that the diet’s inflammatory effect is more pronounced in women, older adults, individuals with high BMI, people with diabetes, and smokers; therefore, it is advisable to implement dietary interventions targeting these high-risk groups, as they are likely to benefit most (Table 6).

**Table 6:** Stratified effects of DII on inflammation by subgroups.

Subgroup	hs-CRP *β*	IL-6 *β*	Interaction *p*
Male	0.17	0.14	0.021
Female	0.22	0.18	0.014
Age <55 yrs	0.15	0.12	0.037
Age ≥55 yrs	0.21	0.16	0.018
BMI < 25	0.11	0.09	0.062
BMI ≥ 25	0.23	0.19	0.008
Diabetics	0.25	0.21	0.006
Non-diabetics	0.14	0.11	0.043
Smokers	0.27	0.23	0.004
Non-smokers	0.12	0.10	0.051

hs-CRP, high-sensitivity C-reactive protein; IL, interleukin; *β*, regression coefficient.

Values represent adjusted longitudinal model estimates across follow-up.

### 3.9 Six-month changes in inflammatory biomarkers by joint DII and PA exposure

The participants who were categorized as having low DII and high PA exhibited the greatest decrease in the levels of inflammation in the body, with Δhs-CRP being −1.18 ± 0.92 mg/L, ΔIL-6 being −1.0 ± 0.7 pg./mL, ΔTNF-*α* being −0.8 ± 0.6 pg./mL, and Δcomposite score being −0.64 ± 0.48 (*p* < 0.001 for all). Participants showing no DII but less PA presented moderate improvements (Δcomposite −0.22 ± 0.41; *p* < 0.001), but those with high DII and high PA experienced only small decreases (Δcomposite −0.09 ± 0.36; *p* = 0.012). It is worth noting that participants with high DII and low PA showed no change or very slight increases in inflammation over the 6 months (Δcomposite +0.12 ± 0.33; *p* = 0.041). Results showed a synergistic effect of diet and exercise, suggesting that switching to an anti-inflammatory diet and increasing PA lead to the greatest reduction in overall inflammation. On the other hand, a diet rich in pro-inflammatory foods, coupled with inactivity, is associated with persistent or mild increases in inflammation, thereby highlighting the necessity of lifestyle changes for the treatment of patients with post-MI (Table 7).

**Table 7:** Change in inflammation over 6 months.

Group	Δhs-CRP	ΔIL-6	ΔTNF-α	ΔComposite	*p*-value
Low DII + High PA	−1.18 ± 0.92	−1.0 ± 0.7	−0.8 ± 0.6	−0.64 ± 0.48	<0.001
Low DII + Low PA	−0.64 ± 0.77	−0.5 ± 0.6	−0.3 ± 0.5	−0.22 ± 0.41	<0.001
High DII + High PA	−0.38 ± 0.69	−0.3 ± 0.5	−0.2 ± 0.4	−0.09 ± 0.36	0.012
High DII + Low PA	−0.11 ± 0.58	−0.1 ± 0.4	0.0 ± 0.3	+0.12 ± 0.33	0.041

Δ indicates change from baseline to 6 months. The composite score represents a standardized systemic inflammation index.

### 3.10 Secondary clinical outcomes at 6-month follow-up

**Figure 5:**
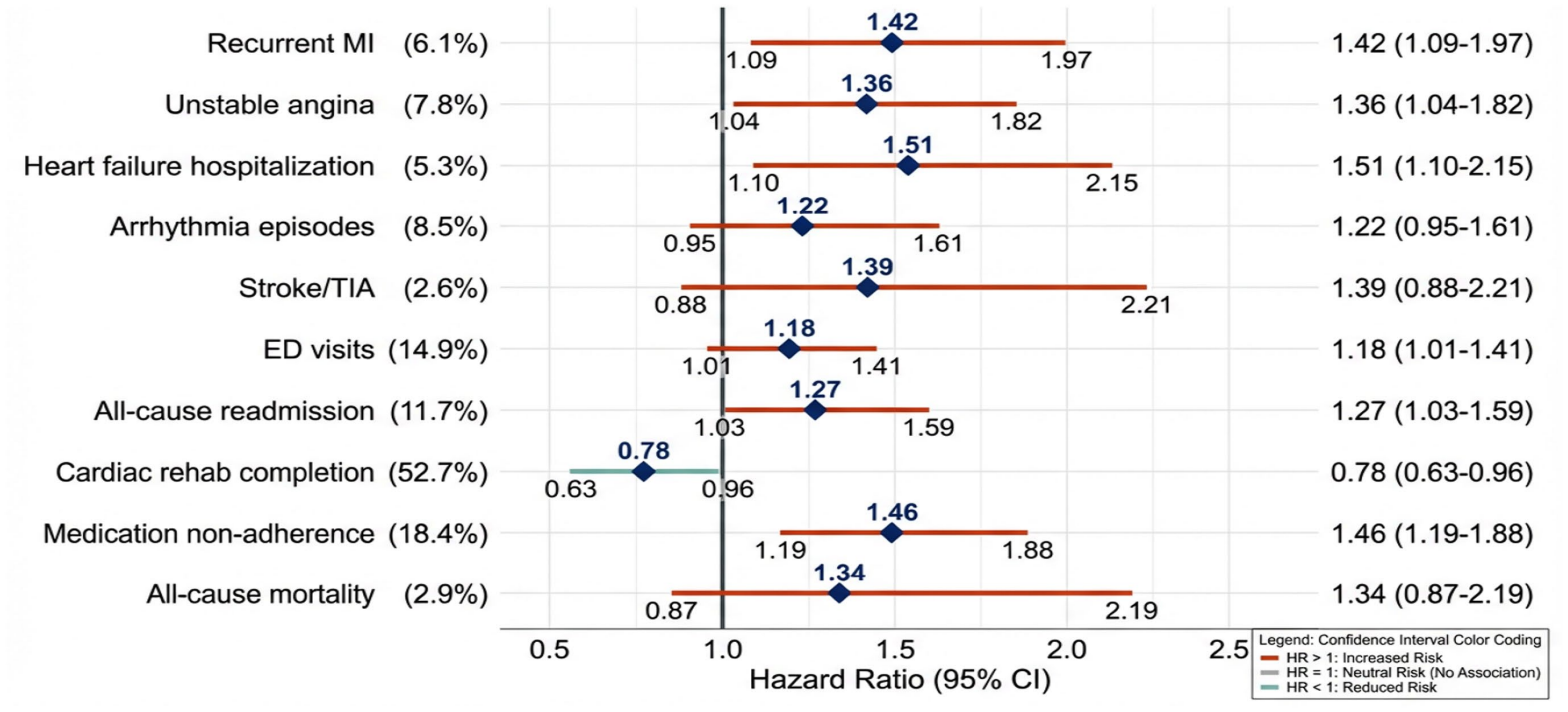
Forest plot of secondary clinical outcomes at 6-month follow-up. The values presented are adjusted longitudinal model estimates that reflect changes from baseline to the 6-month follow-up. ED visits: Emergency Department visits.

Recurrent MI was reported in 6.1% of the total and was associated with a 42% higher risk (HR = 1.42; 95% CI: 1.09–1.97; *p* = 0.019). The unstable angina and heart failure required hospitalization in 7.8 and 5.3% of the patients, respectively, with a significant risk increase (HR = 1.36; 95% CI: 1.04–1.82; *p* = 0.024 for angina; HR = 1.51; 95% CI: 1.10–2.15; *p* = 0.013 for heart failure). Although arrhythmia episodes (8.5%), stroke/transient ischemic attack (2.6%), and all-cause mortality (2.9%) did not reach statistical significance, they exhibited some trends indicating higher risk. The increase in emergency department visits (14.9%) and all-cause readmissions (11.7%) was substantial, with HRs of 1.18 (95% CI: 1.01–1.41; *p* = 0.041) and 1.27 (95% CI: 1.03–1.59; *p* = 0.028), respectively. The correlate of cardiac rehabilitation completion was a 22% lower probability of rescue (HR = 0.78; 95% CI: 0.63–0.96; *p* = 0.019). On the other hand, an 18.4% share of patients who did not comply with their medication was associated with a 46% increase in risk (HR = 1.46; 95% CI: 1.19–1.88; *p* = 0.002). In summary, the studies highlight the significant clinical importance of adherence to lifestyle changes, medications, and rehabilitation programs in preventing secondary cardiovascular events and readmissions among post-MI patients (Figure 5).

### 3.11 Sensitivity analyses for the association between DII, PA, and inflammation

**Figure 6:**
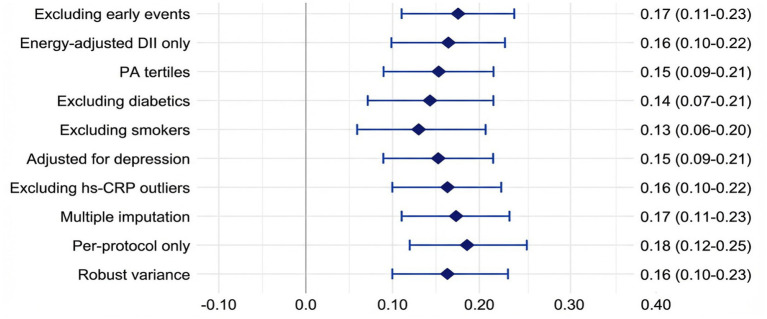
Forest plot of sensitivity analyses for the association between DII, PA, and inflammation.

The researchers performed sensitivity analyses through three methods, which included (1) excluding patients who lacked follow-up information, (2) adding new confounding factors, and (3) dividing participants into age and sex groups. The findings remained consistent across all testing methods, underscoring the robustness of the research outcomes. The association between high DII and inflammation was statistically significant in all models used in the analysis. The exclusion of early events led to an estimate of 0.17 (95% CI: 0.11–0.23; *p* < 0.001), whereas only the energy-adjusted DII analyses yielded a close estimate of 0.16 (95% CI: 0.10–0.22; *p* < 0.001). Splitting PA into tertiles, excluding patients with diabetes, or excluding smokers did not significantly affect the results; estimates remained between 0.13 and 0.15 (all *p* < 0.001). Furthermore, analyses that accounted for depression and removed hs-CRP outliers all yielded significant results (estimates 0.15–0.16; *p* < 0.001). Similarly, per-protocol analyses and models using robust variance estimation yielded the same results (estimates of 0.16–0.18; *p* < 0.001), indicating the stability of the diet- and PA-related effects observed on systemic inflammation. These sensitivity analyses collectively confirm that the relationships among higher dietary inflammatory potential, lower PA, and elevated inflammation remain valid and robust across analytical approaches (Figure 6).

## 4 Discussion

This retrospective cohort trial comprising 1,700 post-MI patients analyzed the independent and synergistic effects of dietary inflammatory potential, assessed via the Dietary Inflammatory Index (DII), and PA compliance on systemic inflammation. Specifically, these results clarified the situation: The patients characterized by low DII values along with high PA compliance presented the most favorable inflammatory signals; on the contrary, those classified with high DII values together with low PA compliance showed the highest levels of hs-CRP, IL-6, TNF-*α*, and composite inflammation scores over a half-year period (22).

High DII, a pro-inflammatory diet, closely correlates with elevated inflammatory biomarkers. This positive relationship is consistent with earlier studies conducted on both general and cardiovascular populations (22–25). By including a post-MI cohort in their studies, the authors show that systemic inflammation is significantly impacted by dietary composition even after an acute coronary event (26–28). The authors found that hs-CRP and IL-6 levels were higher with the intake of saturated fats, refined carbohydrates, added sugars, and sodium; in contrast, consumption of fruits, vegetables, and fibers, as well as omega-3 fatty acids, was protective. This confirms mechanistic evidence that consumption of pro-inflammatory diets drives oxidative stress, leading to endothelial dysfunction and activation of nuclear factor-kappa B pathways, which in turn exacerbate the post-MI inflammatory burden (29, 30).

In line with earlier studies, the level of inflammatory mast cells in the body was found to be negatively correlated with the degree of PA participation (31). Patients who engaged in moderate-to-vigorous PA, walking, and cardiac rehabilitation had lower levels of hs-CRP, IL-6, and TNF-*α*. These findings support the conclusions of previous studies that have established a link between regular PA and decreased systemic inflammation via the above-mentioned mechanisms (32–34). Importantly, our study emphasizes that adherence to PA post-MI can lead to observable anti-inflammatory effects even within a six-month follow-up period, thereby firmly placing it within the realm of secondary prevention strategies.

This study extends the existing literature by examining dietary inflammatory potential alongside PA in a large cohort of patients post-MI. Our research assessed the combined and interactive effects of these factors on systemic inflammation using a long-term, comprehensive clinical data set, thereby providing a comprehensive understanding of how different lifestyles affect disease risk.

One of the major implications of our research is the interplay of DII and PA. Composite inflammation scores were lowest among patients with both low DII and high PA adherence and highest among patients with both high DII and low PA adherence (23, 35, 36). The synergy described above implies that the lifestyle factors are not operating independently but rather work together to achieve the maximum anti-inflammatory effect when both dietary and exercise habits are good. The findings are consistent with earlier studies from community-based and cardiometabolic populations, which have shown that combined diet and PA interventions can result in additive or even multiplicative reductions in systemic inflammation (37–39). From a clinical perspective, these findings underscore the necessity of integrated lifestyle counseling after MI, with an emphasis on combined modifications of diet and PA to maximize cardiovascular recovery and reduce the risk of recurrence (40, 41). The baseline socioeconomic and cardiovascular risk differences between high- and low-DII groups create residual confounding that multivariable adjustment cannot resolve. Thus, all causal inferences need to be understood as provisional findings.

In addition to the inflammatory markers, we observed trends favoring cardiometabolic factors among participants with the lowest DII and highest PA adherence. In this group, systolic and diastolic blood pressure, fasting glucose, HbA1c, LDL-C, and triglycerides improved significantly over 6 months. In contrast, patients with high DII and low PA showed little improvement, or even deterioration, in the parameters. These observations further affirm that systemic inflammation is a marker and mediator of cardiometabolic dysfunction, and that lifestyle factors can concurrently improve biochemical and hemodynamic outcomes (42, 43).

The current study has several strengths. First of all, the large sample size (*N* = 1,700) provides the study with greater statistical power and makes it more representative of the general population. Secondly, the biomarker measurements, repeated every 30 days for 6 months, provided a highly detailed longitudinal assessment of the inflammatory trajectories. Third, the simultaneous consideration of both dietary inflammatory potential and PA adherence gives new insights into the combined lifestyle effects, which are often neglected in post-MI research. The control for many confounders, including medication adherence and participation in cardiac rehabilitation, enhances the credibility of these results. The research demonstrates that post-MI patients experience reduced systemic inflammation when they follow an anti-inflammatory diet and maintain regular exercise. The research demonstrates that lifestyle factors interact to produce clinical results superior to those achieved through independent functioning.

As a retrospective cohort study, it is hard to draw cause-and-effect relationships, and one cannot exclude the possibility of residual confounding by unmeasured variables (e.g., psychosocial stress, sleep quality). Dietary intake was measured using self-reported food frequency questionnaires, which can introduce recall bias. Likewise, PA adherence was self-reported and therefore may have overestimated actual activity levels. Finally, although biomarkers do provide objective measures of systemic inflammation, they capture only overall processes in the body, not tissue-specific ones that may contribute to post-MI remodeling.

The study’s results indicate that after MI management, the dietary inflammatory potential and PA adherence should be routinely assessed as part of secondary prevention. If both diet and exercise are targeted, the resulting anti-inflammatory benefits might be greater than if only one behavior were changed. To confirm these findings, particularly the unexplored mechanisms underlying lifestyle-mediated suppression of systemic inflammation, future prospective and interventional studies are necessary. The research should consider whether improvements in DII and PA adherence are associated with reductions in long-term cardiovascular events, hospitalizations, and mortality.

## 5 Conclusion

This study reveals that dietary inflammatory potential and PA adherence are both independent and synergistic predictors of systemic inflammation in patients after MI. Patients following the anti-inflammatory diet, along with PA, showed the lowest inflammatory burden and the best cardiometabolic profiles after 6 months. These results highlight the necessity of combined lifestyle changes in post-MI treatment and provide practical targets for improving cardiovascular outcomes.

## Data Availability

The raw data supporting the conclusions of this article will be made available by the authors, without undue reservation.
